# Supplementary figures and images for: Production and purification of mannan oligosaccharide with epithelial tight junction enhancing activity
Source: PeerJ. 2019 Jul 2;7:e7206. doi: 10.7717/peerj.7206 (PMC6611449; doi:10.7717/peerj.7206)

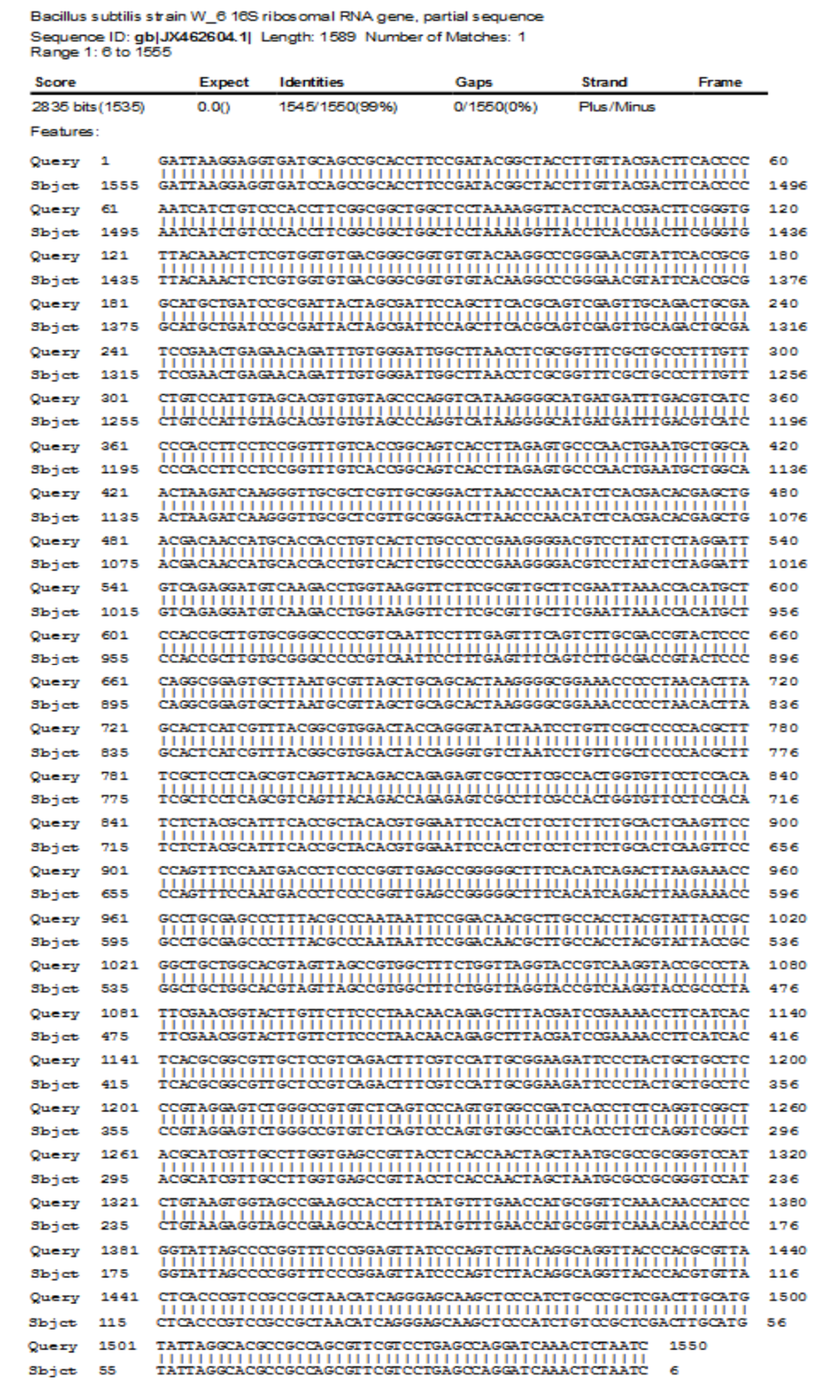

Supplement: Figure S1 [file peerj-07-7206-s001.png]

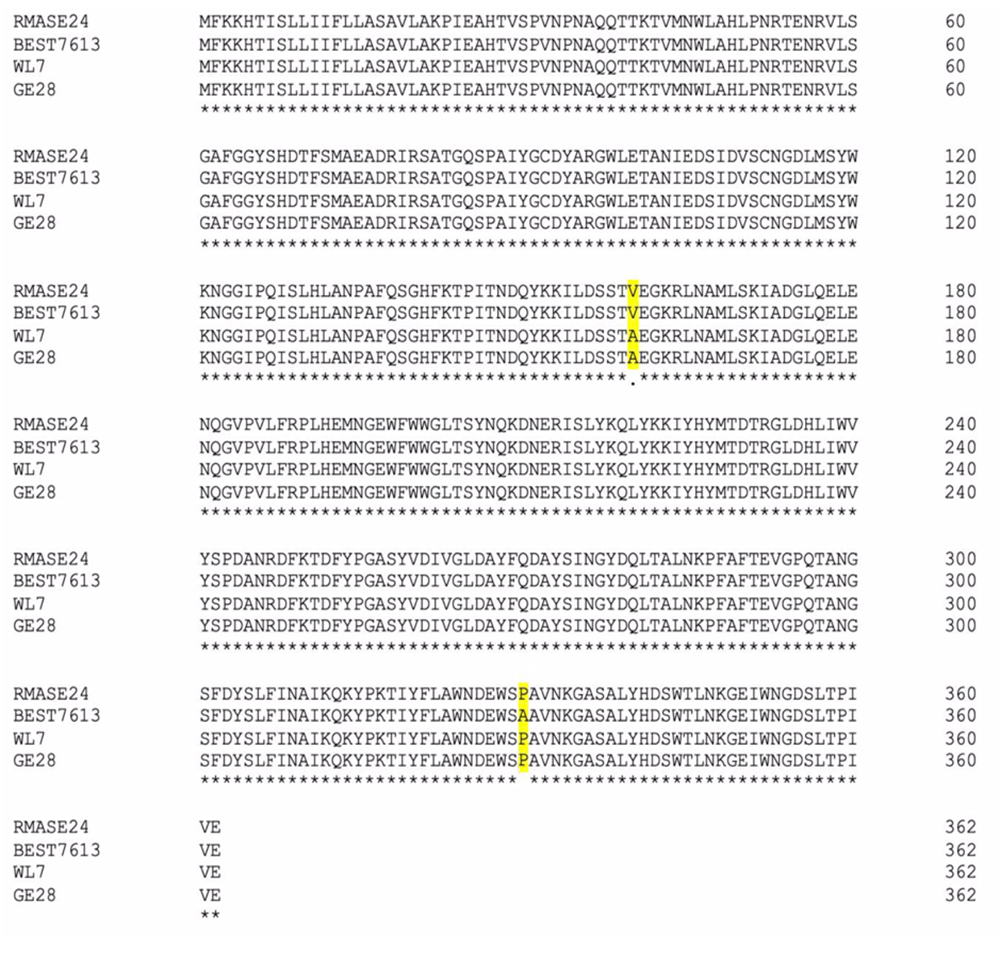

Supplement: Figure S2 — RMASE24 represents the protein sequence of RMase24. BEST7613, WL7, and GE28 represent the protein sequences of mannanase produced from Bacillus subtilis strain BEST 7613, WL7, and GE28 strains. [file peerj-07-7206-s002.png]

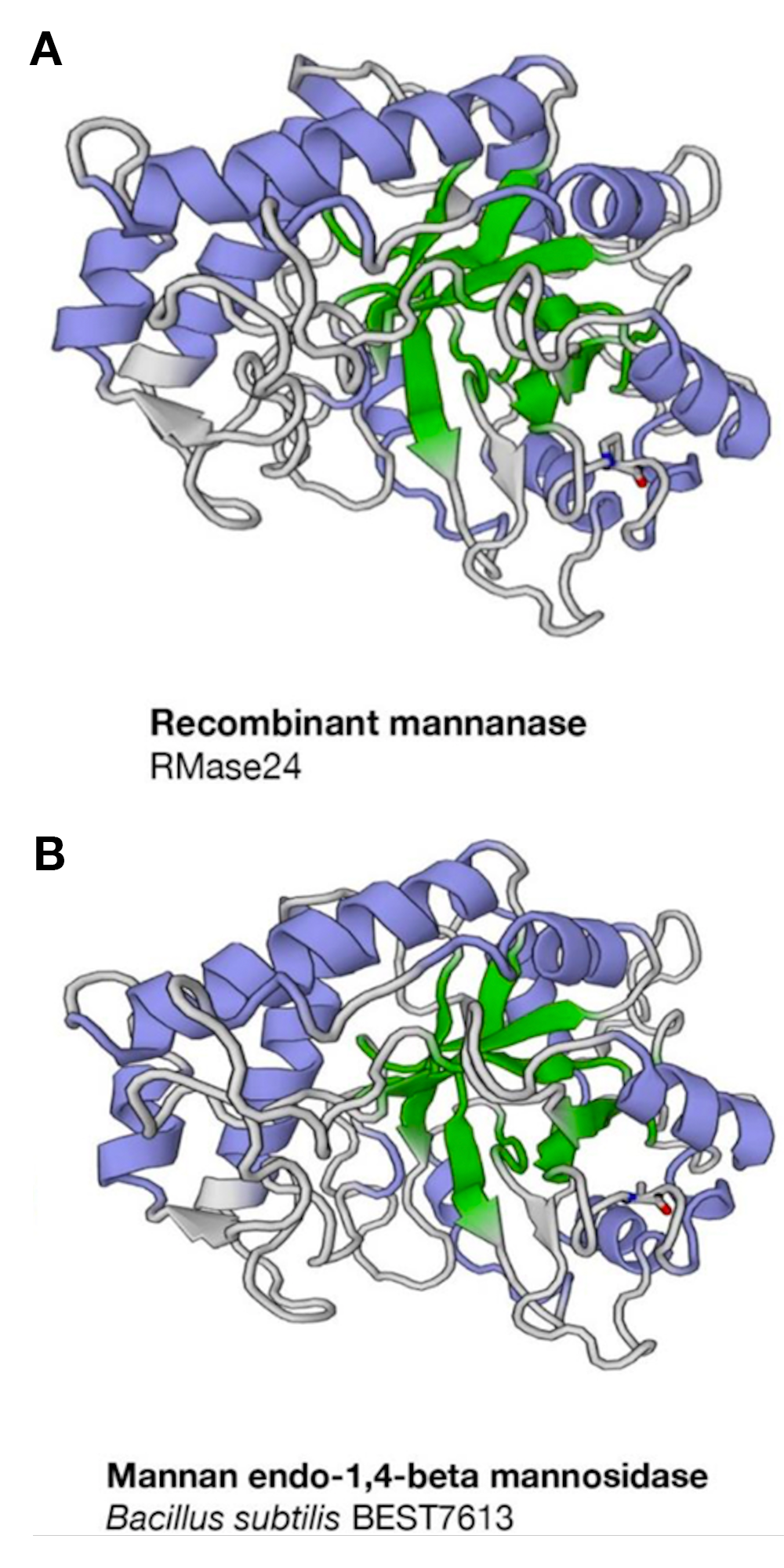

Supplement: Figure S3 — (A) Three-dimentional structure of RMase24. (B) Three-dimentional structure of mannan-endo-1,4-beta-mannosidase from Bacillus subtilis BEST7613. [file peerj-07-7206-s003.png]
