# Supplementary material for: Production and purification of mannan oligosaccharide with epithelial tight junction enhancing activity
Source: PeerJ. 2019 Jul 2;7:e7206. doi: 10.7717/peerj.7206 (PMC6611449; doi:10.7717/peerj.7206)
Supplement: Data S1 [file peerj-07-7206-s004.docx]

GATTAAGGAGGTGATGCAGCCGCACCTTCCGATACGGCTACCTTGTTACGACTTCACCCCAATC ATCTGTCCCACCTTCGGCGGCTGGCTCCTAAAAGGTTACCTCACCGACTTCGGGTGTTACAAAC TCTCGTGGTGTGACGGGCGGTGTGTACAAGGCCCGGGAACGTATTCACCGCGGCATGCTGATC CGCGATTACTAGCGATTCCAGCTTCACGCAGTCGAGTTGCAGACTGCGATCCGAACTGAGAACA GATTTGTGGGATTGGCTTAACCTCGCGGTTTCGCTGCCCTTTGTTCTGTCCATTGTAGCACGTG TGTAGCCCAGGTCATAAGGGGCATGATGATTTGACGTCATCCCCACCTTCCTCCGGTTTGTCAC CGGCAGTCACCTTAGAGTGCCCAACTGAATGCTGGCAACTAAGATCAAGGGTTGCGCTCGTTGC GGGACTTAACCCAACATCTCACGACACGAGCTGACGACAACCATGCACCACCTGTCACTCTGCC CCCGAAGGGGACGTCCTATCTCTAGGATTGTCAGAGGATGTCAAGACCTGGTAAGGTTCTTCGC GTTGCTTCGAATTAAACCACATGCTCCACCGCTTGTGCGGGCCCCCGTCAATTCCTTTGAGTTT CAGTCTTGCGACCGTACTCCCCAGGCGGAGTGCTTAATGCGTTAGCTGCAGCACTAAGGGGCG GAAACCCCCTAACACTTAGCACTCATCGTTTACGGCGTGGACTACCAGGGTATCTAATCCTGTT CGCTCCCCACGCTTTCGCTCCTCAGCGTCAGTTACAGACCAGAGAGTCGCCTTCGCCACTGGTG TTCCTCCACATCTCTACGCATTTCACCGCTACACGTGGAATTCCACTCTCCTCTTCTGCACTCAA GTTCCCCAGTTTCCAATGACCCTCCCCGGTTGAGCCGGGGGCTTTCACATCAGACTTAAGAAAC CGCCTGCGAGCCCTTTACGCCCAATAATTCCGGACAACGCTTGCCACCTACGTATTACCGCGGC TGCTGGCACGTAGTTAGCCGTGGCTTTCTGGTTAGGTACCGTCAAGGTACCGCCCTATTCGAAC GGTACTTGTTCTTCCCTAACAACAGAGCTTTACGATCCGAAAACCTTCATCACTCACGCGGCGT TGCTCCGTCAGACTTTCGTCCATTGCGGAAGATTCCCTACTGCTGCCTCCCGTAGGAGTCTGGG CCGTGTCTCAGTCCCAGTGTGGCCGATCACCCTCTCAGGTCGGCTACGCATCGTTGCCTTGGTG AGCCGTTACCTCACCAACTAGCTAATGCGCCGCGGGTCCATCTGTAAGTGGTAGCCGAAGCCAC CTTTTATGTTTGAACCATGCGGTTCAAACAACCATCCGGTATTAGCCCCGGTTTCCCGGAGTTA TCCCAGTCTTACAGGCAGGTTACCCACGCGTTACTCACCCGTCCGCCGCTAACATCAGGGAGCA AGCTCCCATCTGCCCGCTCGACTTGCATGTATTAGGCACGCCGCCAGCGTTCGTCCTGAGCCAG GATCAAACTCTAATCACTAGT
