# Supplementary material for: Production and purification of mannan oligosaccharide with epithelial tight junction enhancing activity
Source: PeerJ. 2019 Jul 2;7:e7206. doi: 10.7717/peerj.7206 (PMC6611449; doi:10.7717/peerj.7206)
Supplement: Data S2 — The gene sequence of RMase24 was 1,090 bp in size. [file peerj-07-7206-s005.docx]

**Bacillus subtilis recombinant endo 1,4 beta mannosidase gene, complete cds**

>KY951415.1 Bacillus subtilis recombinant endo 1,4 beta mannosidase gene, complete cds

ATGTTTAAGAAACATACGATCTCTTTGCTCATTATATTTTTACTTGCGTCTGCTGTTTTAGCAAAACCAA

TTGAAGCGCATACTGTGTCGCCTGTGAATCCTAATGCCCAGCAGACAACAAAAACAGTGATGAACTGGCT

TGCGCACCTGCCGAACCGAACGGAAAACAGAGTCCTTTCCGGAGCGTTCGGAGGTTACAGTCATGACACA

TTTTCTATGGCTGAGGCTGATAGAATCCGAAGCGCCACCGGGCAATCGCCTGCTATTTATGGCTGCGATT

ATGCCAGAGGATGGCTTGAAACAGCAAATATTGAAGATTCAATAGATGTAAGCTGCAACGGCGATTTAAT

GTCGTATTGGAAAAATGGCGGAATTCCGCAAATCAGTTTGCACCTGGCGAACCCTGCTTTTCAGTCAGGG

CATTTTAAAACACCGATTACAAATGATCAGTATAAAAAAATACTAGATTCTTCAACAGTAGAAGGAAAGC

GGCTAAATGCCATGCTCAGCAAAATTGCTGACGGACTTCAAGAGTTGGAGAACCAAGGTGTGCCTGTTCT

GTTCAGGCCGCTGCATGAAATGAACGGTGAATGGTTTTGGTGGGGACTTACATCATATAATCAAAAGGAT

AATGAAAGAATCTCTCTATATAAACAGCTCTACAAGAAAATCTATCATTATATGACCGACACAAGAGGAC

TTGATCATTTGATCTGGGTTTACTCTCCCGACGCCAACCGAGATTTTAAAACTGATTTTTACCCGGGCGC

GTCTTACGTGGATATTGTCGGATTAGATGCGTATTTTCAAGATGCCTACTCGATCAATGGATACGATCAG

CTAACAGCGCTTAATAAACCATTTGCTTTTACAGAAGTTGGCCCGCAAACAGCAAACGGCAGCTTCGATT

ACAGCCTGTTCATCAATGCAATAAAACAAAAATATCCTAAAACCATTTACTTCCTCGCATGGAATGATGA

ATGGAGCCCAGCAGTAAACAAGGGTGCTTCAGCTTTATATCATGACAGCTGGACACTCAATAAGGGAGAA

ATATGGAATGGCGATTCTTTAACGCCAATCGTTGAATGAA
